# Improved couple satisfaction and communication with marriage and relationship programs: are there gender differences?—a systematic review and meta-analysis

**DOI:** 10.1186/s13643-021-01719-0

**Published:** 2021-06-21

**Authors:** Zeinab Javadivala, Hamid Allahverdipour, Mohammad Asghari Jafarabadi, Somaye Azimi, Neda Gilani, Vijay Kumar Chattu

**Affiliations:** 1 Department of Health Education and Promotion, Tabriz University of Medical Sciences, Tabriz, 14711 Iran; 2 Research Center of Psychiatry and Behavioral Sciences, Department of Health Education and Promotion, Tabriz University of Medical Sciences, Tabriz, 14711 Iran; 3 Department of Statistics and Epidemiology, School of Medicine, Zanjan University of Medical Sciences, Zanjan, Iran; 4 Center for the Development of Interdisciplinary Research in Islamic Sciences and Health Sciences, Tabriz University of Medical Sciences, Tabriz, Iran; 5 Department of Statistics and Epidemiology, Tabriz University of Medical Sciences, Tabriz, Iran; 6 Department of Medicine, Temerty Faculty of Medicine, University of Toronto, Toronto, ON Canada

**Keywords:** Couple satisfaction, Marriage programs, Relationship programs, Systematic review and meta-analysis

## Abstract

**Background:**

The aspects of marriage and relationship and their effect on couples’ satisfaction are essential and critical aspects to be explored in this globalized and contemporary world. Since there are no reported meta-analysis and systematic reviews conducted in the last two decades in this area, we aimed to investigate the effect of marriage and relationship programs (MRP) on couples’ relationship satisfaction (CRS) and couples’ relationship communication (CRC) and also to determine the gender differences if any.

**Method:**

In this systematic review and meta-analysis, the randomized clinical trials (RCTs) published between 2000 and July 26, 2019, were retrieved from several online electronic databases such as Medline, Embase, ProQuest, and Cochrane Library. Inclusion and exclusion criteria were developed using the PICO (Population, Intervention, Comparison, Outcome) framework of PRISMA (Preferred Reporting Items for Systematic Reviews and Meta-Analyses). The mean differences (MDs) and 95% confidence intervals (CIs) were calculated. The reported summary statistics were calculated as random effects models based on the heterogeneity between the studies model. Funnel plots and the Egger regression test was used to confirm the presence of any publication bias.

**Results:**

Of the total 12 intervention studies included, five (5) are education/communication skills programs, three (3) enrichment programs, and four (4) therapy programs. The impact of these programs was investigated on CRS and CRC. Therapy programs had a larger effect than other programs (pooled MD: 0.53 (95% CI = 0.35 to 0.71, I^2^ = 71.5% *p* = 0.0001) and had a larger effect size on wives (pooled MD: 0.53 (95% CI 0.25 to 0.80, I^2^ = 74.1% *p* = 0.0001) than husbands RS (pooled MD: 0.26 (95% CI 0.25 to 0.76, I^2^ = 72.4% *p* = 0.0001). In RC (relationship communication) area, the Enhancement programs showed the small to large effect on CRC (pooled MD: 1.31 (95% CI = 0.13 to 2.50, I^2^ = 94.7% *p* = 0.0001)) and educational programs showed small to medium effect (pooled MD: 0.32 (95% CI = 0.13 to 0.50, I^2^ = 74.5% *p* = 0.0001) on women and no effect on men.

**Conclusion:**

Due to the high effect of the therapy programs on CRS and enhancement program on CRC in the current meta-analysis, the priority of their utilizations in interventions, especially by psychologists and mental health professionals, should be emphasized. Therefore, mental health planning in communities to develop MRP and care for couples’ health should be given special attention to men’s health. Due to the high heterogeneity of the results and with scanty literature in this specific domain, we are uncertain about their actual effect. However, well-designed RCTs with a larger sample size would be beneficial in closely examining the effect of MRPs on CRS and CRC.

**Supplementary Information:**

The online version contains supplementary material available at 10.1186/s13643-021-01719-0.

## Background

Globally, one of the most significant transitions in the human life course in almost every country is either denial from marriage and/or delays in marriage with an increase in cohabitation, divorce rate, remaining single, plus a combination of any of these [1]. Among these factors, marital conflict and subsequent divorce can have adverse consequences for the family and the community [2]. Divorce always is painful and damaging, especially for the divorcing parents and children [3]. There is extensive research evidence that suggests that growing up with single parents is associated with an elevated risk of involvement in crime by adolescents and face the most significant barriers to success in school and the workforce [4–6]. As Heintz suggests, many interventions might prevent divorce and toxic marriage duration, such as compassionate support, encouragement and training, assisting through their difficulties, and developing the skills needed to create and maintain happy and successful marriages [3].

Recent studies in this area indicated that married adults could benefit more than single ones [7, 8]. Marriage offers a certain degree of economic and social stability with improved health and greater satisfaction that unmarried adults do not feel [8]. Besides, it was found that married women are happier than those who were single and have more psychological wellbeing [7, 9]. However, studies by Jackson et al indicated the mental health benefits of being married extend equally to men and women [10, 11]. This issue’s importance is seen in the debate over whether men and women differ in their mental health response to change in marital satisfaction and communication. Though the research results were statistically significant in this study, the gender differences in marital satisfaction between wives and husbands were minimal, with wives slightly less satisfied than husbands; however, this dissatisfaction was found to be higher among wives in marital therapy than the wives in the general population [10]. However, there were no significant gender differences among couples in the general population as per the study findings of Jackson et al and Gager et al. [10, 12].

Various types of marriage and relationship program (MRP) currently exist to increase marriages and relationships, including education/communication skills, enrichment, premarital, counseling, and therapy programs [13]. A meta-analysis published in 2005 has shown the effect of MRP on couples’ relationship satisfaction and communication [13]. Also, the recent literature and the meta-analysis by Jackson et al. indicates that there has been a substantial shift in the distribution of power in marital relationships over the last few decades [10]. According to our knowledge, no recent systematic meta-analysis or study has investigated the impact of MRP on couple satisfaction. Although there were some studies conducted between 2000 and 2019, they have investigated/ reviewed only one specific program such as marriage education [14] or focused only on a particular outcome, such as sexual function [15]. Furthermore, as mentioned above, there is a contradiction among moderator variables crucial to practitioners and policymakers, e.g., participants’ gender differences.

Our meta-analysis exercise aimed to conduct a comprehensive review of articles published in the last 20 years that investigated the effect of MRP on couples’ relationship satisfaction (CRS) and couples’ relationship communication (CRC) and also to determine any gender differences.

## Methods

### Search strategy

**Fig. 1:**
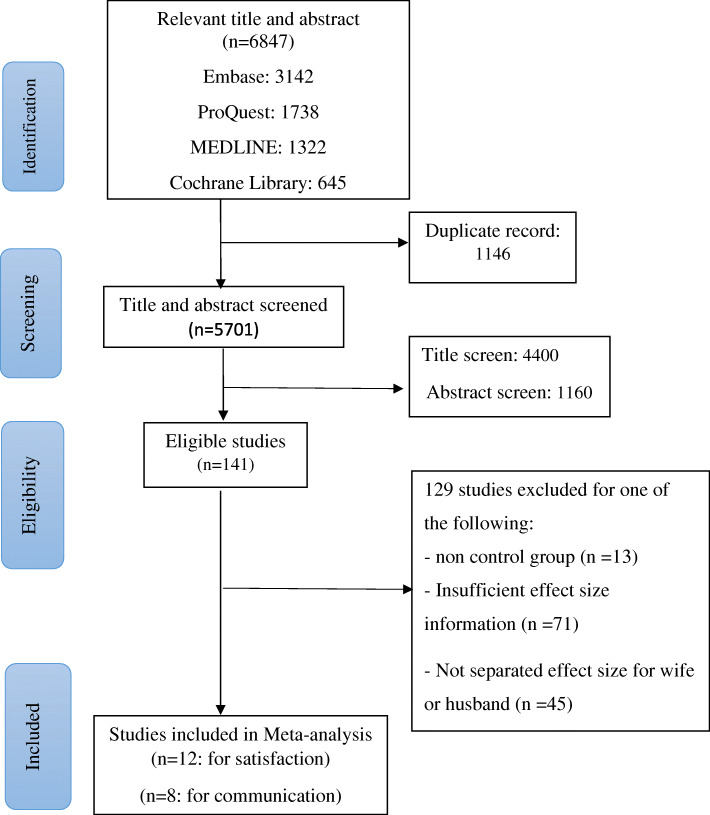
PRISMA flow diagram detailing the search strategy

The current meta-analysis was conducted according to recommendations and standards set by the Preferred Reporting Items for Systematic Reviews and Meta-Analyses (PRISMA) (Supplementary file 1) [16]. A systematic literature review was performed in all major databases for interventional studies that examined the efficacy of MRP interventions and used CRS and/or CRC as an outcome. Both published and unpublished studies were searched by a health sciences librarian in the following databases: MEDLINE, Embase, ProQuest, and Cochrane Library up to July 26, 2019. Besides, a hand search was performed by selecting seemingly relevant articles from the reference list of each included study. To access the concepts of satisfaction, communication, relationship/marriage program, couple and intervention, and numerous text word phrases, using both adjacency operators and truncation to reach the variations in spelling and phrasing, were utilized. Synonymous phrases were first composed with the Boolean “OR”. The five concepts (i.e., satisfaction, communication, relationship/marriage program, couple, and intervention) were then composed with the Boolean “AND”. As detailed in Fig. 1, this search resulted in 5701 non-duplicated records.

### Inclusion criteria

We developed inclusion and exclusion criteria using the PICO framework. *P*opulation: heterosexual couples who had an intimate relationship were included. Couples were excluded if they had an alcohol addiction, a history of suffering from mental illness or cancer and other chronic diseases, being infertile or pregnant; *I*ntervention: marriage and relationship programs. Therapeutic interventions were excluded from providing a clear description of the effects of a psychoeducational intervention. Therapeutic interventions generally have more substantial results than psychoeducational interventions. Studies that aimed to increase sexual functioning were also excluded. *C*omparison: marriage and relationship programs versus no-treatment / waiting list control group; *O*utcomes: measures of CRS and/or CRC with sufficient information to calculate standardized effect sizes and weights.

Studies reported both wife and husband outcome data separately. Studies include randomized control trials (RCTs) design and/or studies using randomization method, random assignment to intervention and control group, and having a control group. The experimental studies were excluded if they had no control group. To be included, a study must have been published between 2000 and 2019 as a full research article and included all the data required to calculate intervention effects with no language barrier.

### Study selection process

The study selection process began with a title and abstract screening by two independent reviewers (ZJ and SA). The selection was based on the inclusion criteria. Articles passing the initial screen were then retrieved and reviewed by ZJ and SA. Reasons for exclusion are listed in Fig. 1. Any disagreements in regard to the selection of articles were resolved by discussion among the four reviewers after reaching an agreement (Zj, HA, MA, and SA). The selected articles were managed by ENDNOTE X9 software. Duplicate studies were excluded. Although two papers were in the Korean language [17, 18], the most important parts, such as tables, were available in English, and therefore the other required contents were translated using Google translator (Google, 2019).

### Outcome measures

We coded measures of relationship quality that assessed two main domains: Relationship satisfaction/dissatisfaction and Relationship communication. All studies (*n* = 12) used standardized measures included the following: Dyadic Adjustment Scale (DAS) [19] (*n* = 4), Couples Satisfaction Index (CSI) [20] (*n* = 2), Enrich Marital Satisfaction Scale (EMSS) [21], Kansas Marital Satisfaction Scale (KMSS) [22], Index of Marital Satisfaction (IMS) [23], Quality of Marriage Index (QMI) [24], Enriching Relationship Issues, Communication and Happiness [21], and Partnership Questionnaire (PQ) [25] for assessing relationship satisfaction (RS). For the area/domain of RC, Communication Skill Test (CST) [26] (*n* = 2), Marital Interaction Coding System (MICS) [27]; Marital Communication Inventory (MCI) [28], Prepare/Enrich Assessment [20] (*n* = 2), Communication Deterioration Factors tool (CDF) [29], and Partnership Questionnaire (PQ) [26] were used.

We examined both immediate post-assessments and follow-up assessments, reporting these separately to reveal improvement (or deterioration) during the time. Timing of follow-up for experimental studies ranged from 3 to 48 months; Einhorn (2010) and Carson (2004) follow-up assessments hold after three (3) months and six (6) months, respectively. Only Haldfor (2000) evaluated the effects of their transition to Self-Regulatory Prevention and Relationship Enhancement Program (Self-PREP) greater than 12 months of intervention at 12 months and 48 months post-assessment. Considerably, most other studies had only post-assessments without any follow-up.

### Data extraction

We classified interventions according to the type of program defined by each article’s author(s), including Enrichment, Education/Communication skills, Counseling and Therapy programs. Other relevant information such as the authors’ names, publication year, the country where the trials were conducted, characteristics of a couple (distressed or un-distressed), study design, type of intervention, the measurement scales, and outcomes were extracted as shown below (Table 1).

**Table 1:** All studies included in the meta-analysis

Serial no, first author, year and country	Participants	Interventions	Durations	Scales	Outcomes
**Education/communication skill programs**					
1. Kroger, 2017 Germany [6]	32 distressed couples	Relationship education program	2 consecutive days	PQ & PQ	Relationship satisfaction and communication skills scores were improved, with moderate to large effects only for soldiers rather than their partners.
2. Li, 2015 China [30]	70 non-distressed couples	Couple relationship education (CRE) programs	Weekends of 2 consecutive weeks	CSI & Enrich	Workshop was effective in improving relationship outcomes, including relationship satisfaction and communication skills.
3.Allen, 2011 United States [26]	476 non-distressed couples	Relationship education for (Prevention and Relationship Education Program	2 days workshop	KMS & CST	Positive intervention effects for satisfaction and communication skills, with sacrificing for the marriage or the partner.
4. Einhorn, 2010 United States [31]	149 distressed couples	Relationship education program	3 Saturday workshops	DAS & CST	The workshop helped improve positive bonding and communication skills for our sample
5. Alvaro, 2001 United States [32]	46 distressed couples	A forgiveness intervention	1-day seminar	EMSS & Enrich	Results suggested the intervention was efficacious in relationship satisfaction and communication skills.
**Enhancement**					
6.Young-Ran, 2012 Korea [17]	16 non-distressed couples	Marital relationship enrichment program	6 weeks	IMS & MCI	Marital satisfaction and communication skills increased significantly after the program in the experimental group compared with the control group.
7.Kong, 2005 Korea [18]	70 non-distressed couples	Marital relationship enhancement program (MREP)	5 weeks	MSS & CDF	Participants in the experimental group showed significant improvements in marital satisfaction and communication skills compared to the control group.
8.Halford, 2000 Australia [33]	83 non-distressed couples	Self-Regulatory Prevention and Relationship Enhancement Program	5 weeks	DAS	High-risk couples receiving Self-PREP exhibited higher relationship satisfaction at 4 years than control couples.
**Therapy**					
9. Doss, 2016 United States [34]	300 distressed couples	Integrative behavioral couple therapy (IBCT)	Eight-hour online program	CSI	Compared to the waitlist group, intervention couples reported significant improvements in relationship satisfaction.
10. Hrapczynski, 2008 United States [35]	50 non-distressed couples	Cognitive behavioral couple therapy (CBCT)	10 weeks	DAS & MICS	Increased relationship satisfaction and communication skills were shown in intervention compared to the control group.
11. Carson, 2004 United States [36]	44 non-distressed couples	Mindfulness-based relationship enhancement	8 weeks	QMI	Results suggested the intervention was efficacious in (a) favorably impacting couples’ levels of relationship satisfaction.
12. Shapo, 2003 United States [37]	43 distressed couples	Support-focused marital therapy (SFMT)	12 weeks	DAS	The SFMT group experienced significantly greater improvements in Marital satisfaction as compared with the control group.

*DAS* Dyadic Adjustment Scale, *CSI* Couples Satisfaction Index, *MSS* Marital Satisfaction Scale, *KMSS* Kansas Marital Satisfaction Scale, *IMS* Index of Marital Satisfaction, *QMI* Quality of Marriage Index, *PQ* Enriching Relationship Issues, Communication and Happiness, and Partnership Questionnaire for the area of satisfaction, *CSI* Communication Skill Index, *CST* Communication Skill Test, *MICS* Marital Interaction Coding System, *MCI* Marital Communication Inventory, *CDF* Prepare/Enrich Assessment and Communication Deterioration Factors tool for the area of communication skills

### Risk of bias

**Fig. 2:**
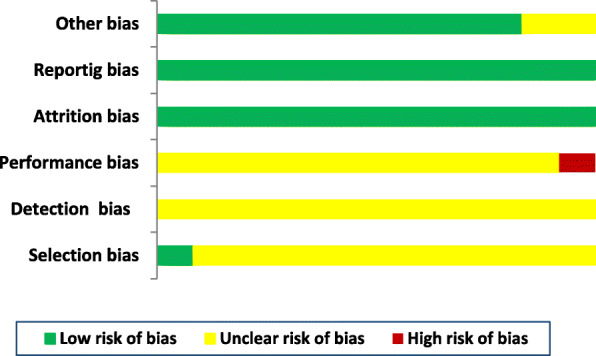
Risk of bias assessment across the studies (*n* = 12)

The risk of bias was judged using the Cochrane Collaboration tool [38], which included six domains, as shown in Fig. 2. Accordingly, each domain was assessed as having a low, unclear, or high risk of bias.

### Generation of effect sizes and data analysis

The mean differences (MDs) and 95% confidence intervals (CIs) were calculated using Stata Corp Stata 16 software. The MD divided by the study’s standard deviation was used to create an index, the standardized MD that would be comparable across the studies. This is the approach suggested by Cohen [39] to describe the magnitude of statistical power analysis effects.

All the studies had provided quantitative data and the weighted mean difference with 95% CI. Heterogeneity was assessed through I squared (I^2^) statistics, and the criterion of significance was I^2^ > 50%. The reported summary statistics were calculated as random effects models based on heterogeneity between the studies model. The chi-square test for heterogeneity was performed to determine whether the results’ distribution was compatible with the assumption that inter-trial differences were attributable to chance variation alone. The level of statistical significance was set at 0.05 a priori. The presence of publication bias was examined using funnel plots and the Egger regression test. To reduce heterogeneity and test the results’ robustness, both the subgroup analysis and sensitivity analysis were performed.

## Results

The literature search from various databases has identified 6847 publications up to July 26, 2019, of which 1146 were excluded as they were duplicates. Around 4400 titles and 1160 abstracts were excluded during the initial screening for the titles and abstracts, and 162 articles were considered for the retrieval of the full texts. Finally, after a detailed assessment, 130 studies were included. An additional eleven studies were obtained by cross-referencing making a final total of 141 studies at this stage. Of the total 141 articles that could be retrieved, thirteen articles were excluded due to a lack of a control group. And among the remaining 116 full-text articles, 71 were excluded due to the lack of statistical information and not receiving email replies from the authors. Additionally, another 45 articles were excluded as they have not reported the effect size for both wife and husband separately (*n* = 45). Finally, 12 articles remained that satisfied all the review criteria and were used for meta-analysis for the relationship satisfaction area, and 8 articles remained for the relationship communication area. Notably, 8 articles contain information for both RS and RC areas while 4 articles had information exclusively for the RS theme (Fig. 1).

### Description of studies

The basic characteristics of the studies are shown in Table 1. According to the type of programs mentioned above, this review contained 76 evaluations of therapy programs, 35 education/communication skills programs, twenty (20) enrichment programs, and ten (10) counseling programs. However, during computation and reporting of effect sizes, among the 12 studies that remained for meta-analysis for RS, five (5) were education/communication skills programs, three (3) enrichment programs, and four (4) therapy programs. Among the eight studies that remained for meta-analysis for RC, five (5) were education/communication skills programs, two (2) were enrichment programs, and the last one being a therapy program. Usually, therapy and counseling programs happen in a clinical setting with a trained psychologist providing treatment. These programs can be based upon a variety of different treatment formats. In this study, the therapy program included were cognitive-behavioral couple therapy (CBCT), Integrative Behavioral Couple Therapy (IBCT) and Support-Focused Marital Therapy (SFMT). Education and communications skills programs tend to be didactic and support both distressed and non-distressed couples. Enrichment programs are usually limited to normal and healthy couples.

Most (*n* = 9) studies were conducted in high-income countries where seven (7) studies are from the USA, one (1) from Germany, and one (1) from Australia. Furthermore, only seven three were conducted in upper- and lower-middle-income countries: 2 from Korea, one (1) from China. The studies included in the meta-analysis had data from 4565 participants (2460 cases and / 2105 controls). All of the entered studies have used randomization methods, random assignment to intervention and control group, and had a control group. However, three (3) studies have reported their study design was a quasi-experimental study [17, 30, 32].

The educational method for Li (2015), Einhorn (2010), and Allen (2011) studies were a weekend workshop; for Alvaro (2001) study, it was held as a seminar, and Doss’s (2016) study ran online calling and chatting. Others used the trained psychologist or trainers for the operation of programs in defined sessions. Kong (2005) and Halford (2000) studies evaluated relationship satisfaction programs with five (5) sessions; Young-Ran (2012), Carson (2004), and Kroger (2017) were between 5 and 10 sessions (6, 8, and 9 sessions perceptively); Hrapczynski (2008) had ten (10) and Shapo (2001) had 12 sessions. For communication skill programs, Alvaro (2001), Allen (2011), and Kroger (2017) have included less than five sessions (4, 4, and 3 sessions perceptively), and Kong (2005), Hrapczynski (2008), Einhorn (2010), Young-Ran (2012), and Li (2015) have held more than five sessions (5, 10, 5, 6, and 6 sessions respectively). Most of the sessions in these studies have lasted for 10 h and above. From the 12 studies, five (5) studies assessed distressed couples, six (6) studies contained non-distressed couples, and one (1) study contained both distressed and non-distressed couples.

### Risk of bias

The risk of bias was assessed for clinical trials. The bias such as the unclear risk of selection bias (due to lack of information on the method of randomization *n* = 11 and concealment, *n* = 12), performance bias (due to lack of information on blinding of participants and personnel, *n* = 12), and detection bias (blinding of outcome assessment, *n* = 12) were observed. The risk of bias for the included studies was low for reporting bias, attrition bias, and other sources of bias (Fig. 2).

### Effects of interventions (meta-analysis results)

Of the total 12 interventions, the impact of five (5) education/communication skills programs, three (3) enrichment programs, and four (4) therapy programs were investigated on RS among couples.

**Fig. 3:**
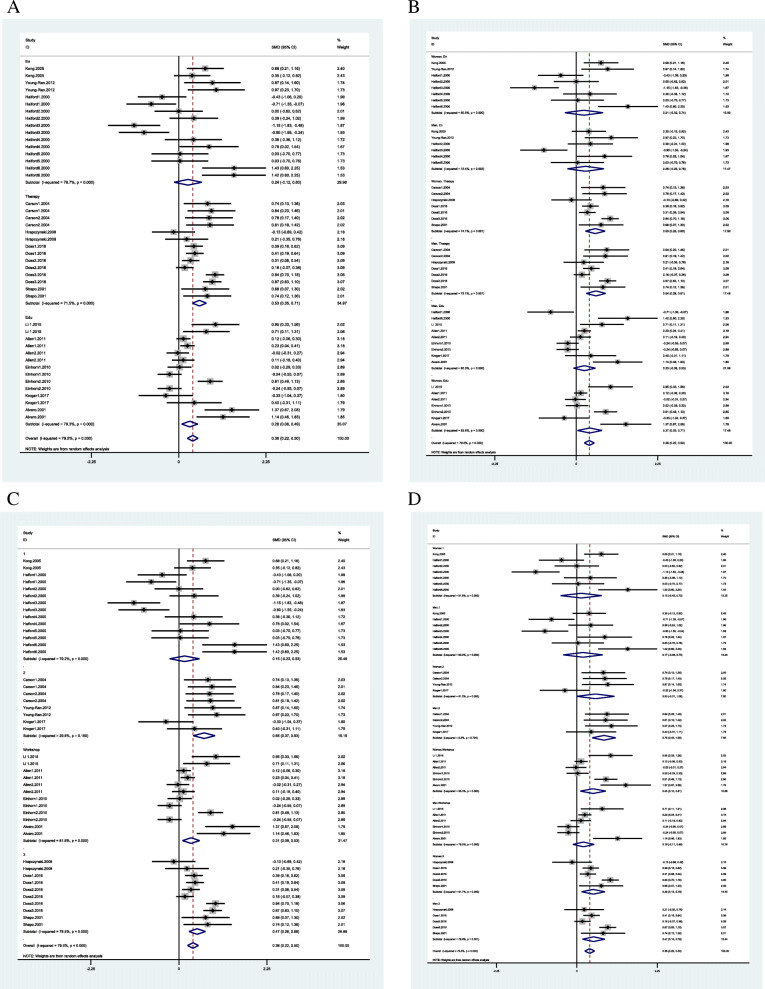
Couple relationship satisfaction subgroup analysis based on type of programs (**A**); gender and type of programs (**B**); number of sessions (**C**: 1: fewer 5 sessions, 2: between 5 and 10 sessions, 3: more than 10 sessions); gender and number of sessions (**D**)

The pooled MD was 0.28 (95% CI = 0.08 to 0.49, I^2^ = 79.3% *p* = 0.0001), 0.24 (95% CI = − 0.12 to 0.60, I^2^ = 78.7%, *p* = 0.855), 0.53 (95% CI = 0.35 to 0.71, I^2^ = 71.5%, *p* < 0.0001 0.000) respectively. As a result, the therapy programs showed the medium to large effect on CRS and educational programs showed small to medium effect. But the heterogeneity among studies was high. Enhancement programs showed no effect on CRS (Fig. 3A). In the area of RC, the enhancement programs showed small to large effect on CRC (pooled MD 1.31 (95% CI = 0.13 to 2.50, I^2^ = 94.7% *p* = 0.0001)) similar to the educational programs which also showed small to medium effect (pooled MD 0.32 (95% CI = 0.13 to 0.50, I^2^ = 74.5% *p* = 0.0001). However, the heterogeneity among these studies was high (Fig. 5A).

### Subgroup analysis

The subgroup analysis conducted for following variables: gender differences (men, women), number of sessions (1: fewer 5 sessions, 2: between 5 and 10 sessions, 3: more than 10 sessions and workshop), total hours of interventions (1: fewer 10 h, 2: between 10 and 15 h, 3: more than 15 h), distress status (distressed and non-distressed), program type (therapy, enrichment, education/communication skills, and counseling). These variables were considered as moderators of marital satisfaction and communication effects based on previous literatures [10, 13, 14]. Subgroup analyses show that effect sizes are different for different subgroups of studies in current study.

**Fig. 4:**
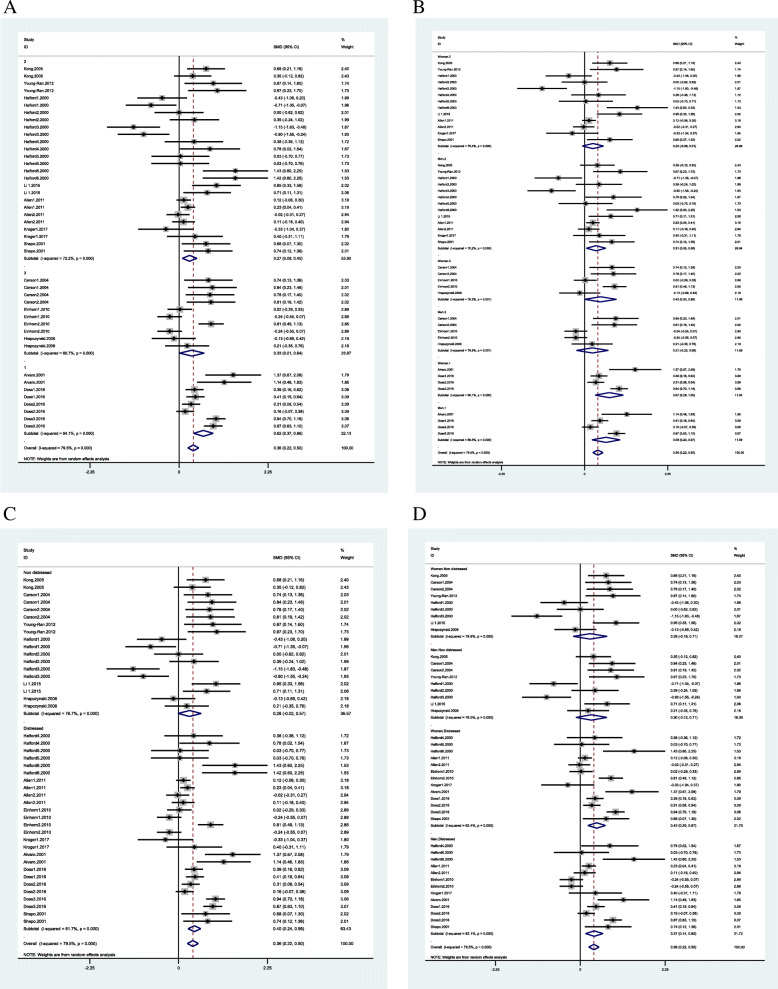
Couple relationship satisfaction subgroup analysis based on number of hours (**A**: 1: fewer 10 h, 2: between 10 and 15 h, 3: more than 15 h); gender and number of hours (**B**); status of distress (**C**); gender and status of distress (**D**)

In the RS area, the gender-based subgroup analysis showed the therapies had a more significant effect size on wives than husbands, and education was only effective for wives and enhancement programs with no effect for both couples (Fig. 3B). The subgroup analysis based on the number of sessions showed the between 5 and 10 sessions had a medium to large effect on CRS, and more than ten (10) sessions and workshops had small to medium effect but with high heterogeneity. Moreover, fewer than five (5) sessions did not affect couples’ relationship satisfaction (Fig. 3C). The subgroup analysis based on both the number of sessions and gender showed between 5 and 10 sessions had a larger effect size on husbands than wives. However, the workshop had no impact, and more than ten (10) sessions had the same effect on husband and wives (Fig. 3D). The subgroup analysis based on total hours of interventions showed fewer than 10 h of intervention had a medium to large effect on CRS while the more than 10 h had a small to medium effect (Fig. 4A). The subgroup analysis based on both hours and gender showed fewer than 10 h of intervention had a larger effect on wives’ relationship satisfaction than husbands. In contrast, between 10 and 15 h of intervention were effective only for husbands (Fig. 4B).

The subgroup analysis based on distressed and non-distressed couples showed interventions had small to medium effects on both distressed and non-distressed couples but with high heterogeneity (Fig. 4C). The subgroup analysis based on both distress/non-distressed and gender showed the interventions had a small to medium effect on both distressed wives and husbands but no effect on both non-distressed wives and husbands (Fig. 4D). As well, subgroup analysis based on distressed level, program type, and gender showed the only therapy programs were effective for both distressed wives and husbands and merely for non-distressed husband with small to larger effect without heterogeneity. Therapies and educational/communication skills were non-effective for both distressed and non-distressed wives and husbands. Sensitivity analysis did not apply to these results due to non-existing outlier data.

**Fig. 5:**
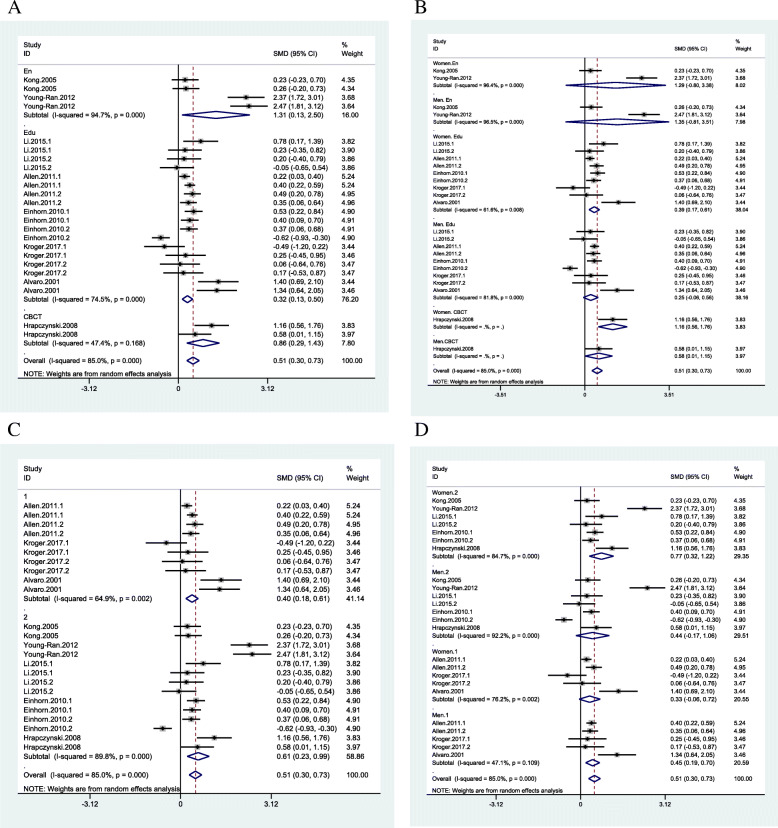
Couple communication skills subgroup analysis based on type of programs (**A**); gender and type of programs (**B**); number of sessions (**C**: 1: fewer 5 sessions 2: between 5 and 10 sessions); gender and number of sessions (**D**)

**Fig. 6:**
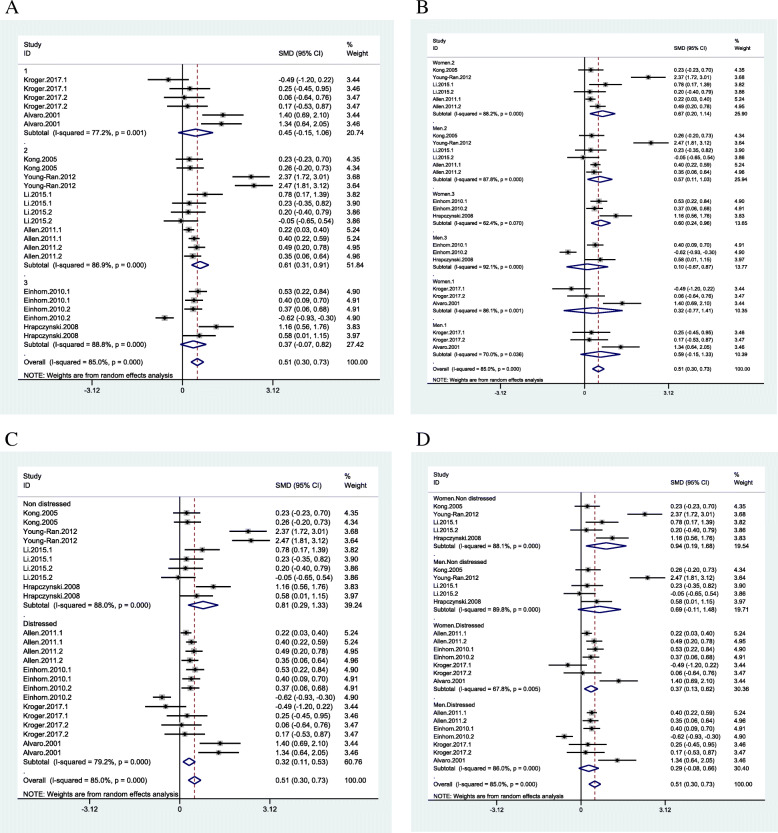
Couple communication skills subgroup analysis based on number of hours (**A**: 1: fewer 10 h, 2: between 10 and 15 h, 3: more than 15 h); gender and number of hours (**B**); status of distress (**C**); gender and status of distress (**D**)

In the RC domain, the gender-based subgroup analysis showed that “education” was effective only for wives (Fig. 5B). The subgroup analysis based on the “number of sessions” showed that more than 5 sessions had a small to medium effect and less than 5 sessions had small to large effect on couples’ relationship communication (CRC) but with high heterogeneity (Fig. 5C). The subgroup analysis based on both the “number of sessions” and “gender” showed that less than 5 sessions had a small to medium effect on husbands with no effect on wives. Conversely, more than 5 sessions had a small to large effect on wives with no effect on husbands (Fig. 5D). The subgroup analysis based on “total hours of interventions” showed a small to large effect on CRC for interventions between 10 and 15 h (Fig. 6A). The subgroup analysis based on both “hours of intervention” and “gender” showed a small to larger effect on both husband and wives’ relationship communication for interventions between 10 and 15 h. In contrast, more than 10 h of intervention were effective only for wives (Fig. 6B).

The subgroup analysis based on “distressed” and “non-distressed” couples showed that interventions had “small to medium” effects on distressed couples and “small to large” effect among non-distressed couples but with high heterogeneity (Fig. 6C). The subgroup analysis based on both “distress/non-distressed” and “gender” showed that the interventions had “small to medium” effect among the distressed wives and “small to large” effect on non-distressed wives with no effect on distressed and non-distressed husbands (Fig. 6D).

### Publication bias

**Fig. 7:**
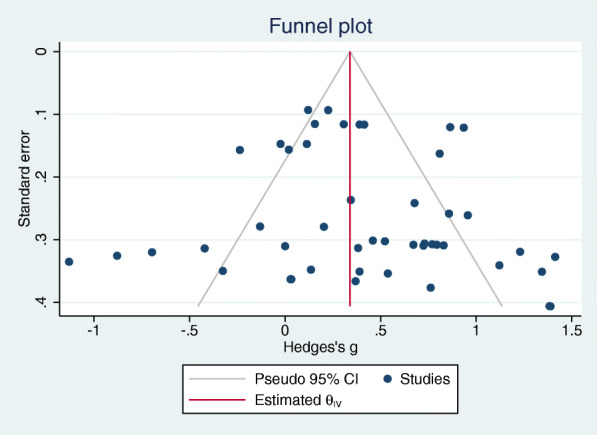
Funnel plot of included interventions

Funnel plot 12 studies showed no evidence suggestive of publication bias, and also, the results of the Egger test were insignificant for publication bias (*p* = 0.460) (Fig. 7).

## Discussion

The purpose of this meta-analysis was to investigate more critically the effects of recently two past decade MRP on CRS and CRC. Our analyses provide simple and clear evidence that MRPs have a “small to moderate” effect size in the overall improvement of both the CRS (0.22 to 0.50) and CRC (0.30 to 0.73). However, in our study it was found that the effect size was similar for both wives and husbands. The previous meta-analysis [13] had shown a moderate to larger effect size (0.54 to 0.82) on CRS while it was found to be small to medium effect on CRC (0.06 to 0.45) without any analysis on the gender differences. These variations may be due to differences in inclusion criteria, types of studies, and differences in included studies’ methodological characteristics. Furthermore, the findings from the vast literature indicate that there has been a substantial shift in the distribution of power in marital relationships over the last few decades [10].

Looking at differences in effect sizes across characteristics of programs discloses some similarities and differences between this study and previous meta-analyses. In our study, while comparing the program types (therapy, enrichment, education/communication skills, and counseling), any study that contained counseling program did not meet our eligibility/inclusion criteria. In contrast, the previous meta-analyses included all program types and counseling had the more effect size. Consistent with Anderson’s study, therapies were most effective compared to education and enrichment programs. The meta-analysis by Hawkins et al. [14] on marriage and relationship education on relationship quality has shown small effect sizes (0.30 to 0.36) similar to our study (0.01 to 0.40). In this study, the type of RC programs was in coherence with Anderson’s study that assessed therapy, enrichment, and education/communication skills for improving CRC. In this meta-analysis, although all program types were effective in improving couples’ communication, in Anderson’s study only education/ communication skills programs were found to be effective with “small to large” effect. Reasons for this difference may be related to the advent of modern techniques in couple therapy compared to the past.

Due to the high effect of the therapy programs on CRS and enhancement program on CRC in the current meta-analysis, the priority of their utilizations in interventions by psychologists and mental health professionals should be emphasized. Besides, our finding showed that therapy programs in improving RS and educational programs for RC are found to be more effective for women than men. The probable reason could be that the dissatisfied women are more likely to go to clinics and seek treatment to improve their condition [10], so that the early treatment may have a greater impact on them compared to men. The meta-analysis by Jackson et al. [10] showed that the dissatisfaction is higher among wives who were referred to marital therapy than the wives in the general population. Also, the wives who were referred to marital therapy were less likely to be satisfied with their marital relationship than the husbands.

Because of the small effect of educational programs on CRS and CRC, the same as the previous meta-analysis [13], future researchers should implement high quality with innovative strategies developed by psychoeducational professionals and researchers for enhancing the effectiveness. In support of the findings from Anderson’s meta-analyses, the enhancement programs were non-effective in CRS but a large effect on CRC. As enhancement programs are usually developed for non-stressed couples, they might not be very serious about leaning programs and practicing with a partner. Moreover, consistent with our study, previous studies showed that MRP was less effective for RS of non-distressed couple samples than the distressed sample [13, 14]. Although this study indicated MRPs are effective on RC of both distressed and non-distressed couple, previous studies showed that MRP had no effect on RC of “non-distressed couple” samples compared to the “distressed couple” sample.

Surprisingly, MRP had more effectiveness for RS of distressed women than did for men and in case of RC of both distressed and non-distressed women, MRP had effectiveness, while it was non-effective for men. Therefore, mental health planning in communities to develop MRP and care for couples’ health should be given special attention to men’s health. Future studies should prioritize special MRPs to improve RS of distressed men and RC of distressed and non-distressed men.

The polled effect size for RS programs with 5 to 10 sessions had a larger effect than programs with more than 10 sessions, while for RC programs with 5 to 10 sessions had a larger effect than programs with less than 5 sessions. Anderson’s study has indicated 5 or more sessions of RS program and RC program are more effective than fewer ones. However, in both studies, less than 10 h of RS and RC programs were more effective than 10 h and above. It seems that in addition to hours and sessions, other factors might be related to increasing effectiveness in MRP. In other words, the longer session or hours might not guarantee the effectiveness of MRP; using beneficial and practical content might be more effective than the number of hours and sessions.

Further studies are needed to examine factors that can enhance the effectiveness of MRP. It is considered that the fewer sessions and hours were more effective for husbands than wives, but with increased sessions and hours, both had the same output in RSP. However, the longer sessions and hours were more effective for wives than husbands in RCP. The other aspect to highlight is that the compact sessions such as seminars and workshops were not effective for the husbands in RSP. That means the husbands might need fewer sessions and fewer hours through consecutive sessions, instead of compact ones such as a workshop, to enhance the RS. Therefore, it would be more interesting if future studies examine the effect of the number of sessions and hours of MRPs between couples and their differences among wives and husbands separately.

In the present study, most studies have been conducted in developed countries such as the USA. Such programs are negligible/ not popular in developing countries due to their poor socio-economic and political conditions. So, providing and supporting opportunities for utilization MRPs and conducting high-quality research in developing countries will play a considerable role in narrowing this gap and increasing their CRS and CRC and family psychological well-being [7, 40].

The level of heterogeneity in our results was high. This finding is not surprising as the reviewed studies were used different scales in different cultures with differences in the sample size. The minimum sample size was estimated to be 29, and the maximum was 461 participants. The duration of interventions varied from a 1-day seminar to 12 weeks. The duration for the majority of the interventions ranged from 5 to 10 weeks. Therefore, the heterogeneity of effect size cannot be linked to the studies’ duration as there is no evidence of an association between the duration of intervention and effect size.

Delimitations and limitations of the study must be acknowledged. Different types of interventional programs and the pooling of various treatments together may be considered as we found other factors for the heterogeneity in our study findings. By applying the random effect model, we have tried to control heterogeneity to account for various studies’ variations. We also conducted a subgroup analysis to decrease heterogeneity and sensitivity analysis to assess the soundness of the results. However, no substantial reduction was detected in the observed heterogeneity. Variation in the tools applied to measure the change in MRP was recognized as a main limiting factor.

Additionally, a few numbers of measures were not validated. The nocebo effect is a substantial problem in psychosocial interventions [41]. In psychosocial studies, the absence of blinding and the failure in adjusting for the nocebo effect in the analysis were regarded as limitations in determining the true intervention effects. Due to the high heterogeneity of the results and with scanty literature and evidence available in these domains, we are uncertain about the actual effect.

## Conclusion

Our finding showed that for women, therapy programs are more effective in improving RS and educational programs for RC than men. Surprisingly, MRP had more effectiveness for RS of distressed women than did for men, and RC of both distressed and non-distressed women, MRP had effectiveness, while, for men, it was non-effective. Therefore, planning mental health for communities to develop MRP, care for couples’ health and men’s health should be given special attention. Due to the high effect of the therapy programs on CRS and enhancement programs on CRC in this meta-analysis, the importance of their utilizations through the interventions, especially by psychologists and mental health professionals, should be emphasized. Furthermore, psychologists and mental health professionals should consider developing programs with fewer sessions and fewer hours through consecutive sessions, instead of compact ones such as workshops to enhance men’s RS. Additional research exploring the gender differences and the gaps between developed and developing countries is warranted as there are very few studies from developing countries. It would also be helpful to examine more closely the long-term effects of MRPs on CRS and CRC through well-designed RCTs with larger sample size. Lastly, it would be important to examine possible gender differences in marital satisfaction and communication among non-heterosexual couples as well.

## Supplementary Information

**Additional file 1.**

## Data Availability

At present, data are not freely available (however available on request) for the reason that we are still writing up another paper for publication.

## Abbreviations

CMRP: Couple Marriage Relationship Program
CRS: Couple Relationship Satisfaction
MRP: Marriage Relationship Program
RCTs: Randomized clinical trials
RC: Relationship communication
RS: Relationship Satisfaction
